# Systemic treatments and outcomes in *CIC* ‐rearranged Sarcoma: A national multi‐centre clinicopathological series and literature review

**DOI:** 10.1002/cam4.4580

**Published:** 2022-02-17

**Authors:** Elizabeth A. Connolly, Vivek A. Bhadri, Johnathon Wake, Katrina M. Ingley, Jeremy Lewin, Susie Bae, Daniel D. Wong, Anne P. Long, David Pryor, Stephen R. Thompson, Madeleine C. Strach, Peter S. Grimison, Annabelle Mahar, Fiona Bonar, Fiona Maclean, Angela Hong

**Affiliations:** ^1^ Chris O'Brien Lifehouse Sydney New South Wales Australia; ^2^ Royal Prince Alfred Hospital Sydney New South Wales Australia; ^3^ Faculty of Medicine and Health The University of Sydney Sydney New South Wales Australia; ^4^ Crown Princess Mary Cancer Centre Westmead Sydney New South Wales Australia; ^5^ Peter MacCallum Cancer Centre Melbourne Victoria Australia; ^6^ ONTrac at Peter Mac Victorian Adolescent & Young Adult Cancer Service; ^7^ Sir Peter MacCallum Department of Oncology The University of Melbourne Melbourne Australia; ^8^ Anatomical Pathology, PathWest QEII Medical Centre Perth Western Australia Australia; ^9^ Sir Charles Gardiner Hospital Perth Western Australia Australia; ^10^ Princess Alexandra Hospital Brisbane Australia; ^11^ Prince of Wales Hospital Sydney New South Wales Australia; ^12^ Faculty of Medicine University of New South Wales Sydney New South Wales Australia; ^13^ NSW Health Pathology Sydney New South Wales Australia; ^14^ Anatomical Pathology, Douglass Hanly Moir Pathology Sonic Healthcare Macquarie Park New South Wales Australia

**Keywords:** CIC, DUX4, ewing‐like sarcoma, rearrangement, round cell sarcoma, ultra‐rare sarcoma

## Abstract

*CIC*‐rearranged sarcoma is a recently established, ultra‐rare, molecularly defined sarcoma subtype. We aimed to further characterise clinical features of *CIC*‐rearranged sarcomas and explore clinical management including systemic treatments and outcomes.

**Methods:**

A multi‐centre retrospective cohort study of patients diagnosed between 2014–2019.

**Results:**

Eighteen patients were identified. The median age was 27 years (range 13–56), 10 patients were male (56%), 11 patients (61%) had localised disease and 7 patients had advanced (metastatic or unresectable) disease at diagnosis. Of 11 patients with localised disease at diagnosis, median overall survival (OS) was 40.6 months and the 1‐, 2‐ and 5‐year OS estimates were 82%, 64% and 34% respectively. Nine patients (82%) underwent surgery (all had R0 resections), 8 (73%) patients received radiotherapy to the primary site (median dose 57Gy in 28 fractions), and 8 (73%) patients received chemotherapy (predominantly Ewing‐based regimens). Metastases developed in 55% with a median time to recurrence of 10.5 months. In patients with advanced disease at diagnosis, median OS was 12.6 months (95% CI 5.1–20.1), 1‐year OS was 57%. Median progression‐free survival was 5.8 months (95% CI 4.5–7.2). Durable systemic therapy responses occurred infrequently with a median duration of systemic treatment response of 2.1 months. One durable complete response of metastatic disease to VDC/IE chemotherapy was seen. Responses to pazopanib (*n* = 1) and pembrolizumab (*n* = 1) were not seen.

**Conclusion:**

In this series, *CIC*‐rearranged sarcomas affected young adults and had a high incidence of presenting with, or developing, metastatic disease. The prognosis overall was poor. In advanced disease, durable systemic therapy responses were infrequent.

## Graphical abstract

Multi‐centre retrospective series of systemic therapy and outcomes for recently established ultra‐rare sarcoma subtype. CIC‐rearranged sarcomas appear to be relatively chemo‐insensitive as illustrated by a high frequency of relapse in localised disease and short durations of treatment response in the advanced setting. In advanced disease, durable systemic therapy responses in CIC‐rearranged sarcoma are limited with a median duration of systemic treatment response of 2.1 months in this series.

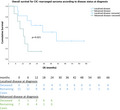

## 1 INTRODUCTION

*CIC‐*rearranged sarcoma is a recently established ultra‐rare^1^ clinically and molecularly distinct subtype of high grade undifferentiated sarcoma, which is defined by *CIC*‐related gene fusions.^2^, ^3^ Due to it's rarity there is a lack of consensus on how to classify and risk stratify this molecular subtype.^4^ *CIC*‐rearranged sarcomas are small round blue cell tumours. Prior to recognition of the entity these were most likely called ‘atypical’ Ewing sarcoma or undifferentiated round cell sarcoma, not otherwise specified. They often present in younger adults (median age 25–35 years). Tumours predominantly arise in soft tissue though can arise in viscera (10%) including brain, and bone (<5%).^3^ A *CIC‐DUX4* fusion is present in 95% of cases, though other *CIC*‐partners exist including *FOX04*, *LEUTX*, *NUTM1* and *NUTM2a*.^2^

Understanding of its natural history, clinical behaviour and treatment outcomes is limited with less than 200 cases reported in the literature and less than 100 cases include clinical follow‐up or treatment information. *CIC‐*rearranged sarcomas appear to follow an aggressive course and are linked to poorer treatment responses and survival outcomes compared to Ewing sarcoma. In the largest published series of 115 cases, of which clinical follow up information was available for 57 cases, the 2‐ and 5‐year overall survival (OS) rates were 53% and 43%.^2^ Comprehensive information on clinical management, in particular systemic treatment use and their outcomes, is scarce.

The aim of this study was to characterise clinical features of *CIC*‐rearranged sarcomas and evaluate clinical management, including systemic treatments and outcomes, through assessment of a series of patients from multiple institutions within Australia.

## 2 MATERIALS AND METHODS

A national multi‐centre retrospective cohort study was undertaken of patients with a diagnosis of *CIC*‐rearranged sarcoma. Patients were included where a diagnosis of *CIC*‐rearranged sarcoma had been performed by pathologists with sarcoma expertise and included fluorescence in situ hybridisation (FISH) confirmation of *CIC*‐rearrangement. Data collection and usage for this study was approved by the Sydney Local Health Human Research Ethics Committee (X17‐0340).

Patient demographics, clinical data, treatment response and outcomes were collected by retrospective record review. Systemic chemotherapy response was assessed by individual sites from the record or radiological review. Time to disease progression was defined as time from first dose of chemotherapy to time of radiological or clinical progression. Overall survival was measured from the time of diagnosis to the date of death and censored at last follow up. Progression‐free survival (PFS) was defined as the time from diagnosis until the date of first progression, death, or censored at last follow up. A median ‘duration of systemic treatment response’ was calculated for those with advanced disease who received systemic treatment as a single modality (without concurrent surgical resection or radiotherapy). This was calculated given a number of patients with advanced disease receive multi‐modality treatment in the first line, which may prolong PFS and limit evaluation of systemic therapy efficacy, and to capture the efficacy of multiple treatments used including those in the second or third line. This was defined as the time from commencing treatment until the date of radiological or clinical progression or censored at the date of last follow up. Median follow‐up was calculated using a reverse Kaplan–Meier method. Survival analysis was completed by the Kaplan–Meier method with comparison of patient groups by log rank method. A *p*‐value of less than 0.05 was considered as statistically significant. Statistical analysis was calculated using IBM SPSS statistics, version 27.

## 3 RESULTS

### 3.1 Patient clinicopathological characteristics

A summary of baseline characteristics is displayed in Table 1. In total, 18 patients, who were diagnosed between 2014 and 2019, were identified across 6 Australian institutions. The median age at diagnosis was 27 years (range 13–56) and 10 (56%) patients were male. Patients were aged between 20 and 40 years in 14 cases (78%). At diagnosis, 11 patients (61%) had localised disease and 7 patients had advanced (metastatic or unresectable) disease. Primary sites included soft tissue in 14 (78%) patients, visceral in 3 (17%; pleural, frontal lobe of brain without involvement of dura or skull) and bone in 1 (6%; ilium) with a median primary tumour size of 63 mm (range 27–150). Metastatic disease sites, at diagnosis and throughout disease, included lung, liver, lymph nodes, brain and bone.

**TABLE 1:** Clinical, treatment and outcome details of 19 CIC‐rearranged sarcoma diagnosed in Australia between 2014 and 2019

Case	Age/sex	Primary	Size (mm)	Surgery, margin	Primary Site Radiotherapy (dose, fraction)	Systemic Rx for Localised disease	Systemic Rx for Advanced disease	Sites of all metastases during disease	Disease Status	Follow up time or survival time from date of diagnosis (months)
Localised disease at diagnosis										
1	38 M	Supraclavicular mass	56	Y, 1 mm	60 Gy, 30#	—	AC, GD, IT	Lung, T4/5 soft tissue	Local & distant R at 10 m, DOD	29.3
2	23F	Psoas	113	N	50 Gy, 25#	VDC/IE then VCDE^a^	Nil (rapid progression)	Lung, liver	Local & distant R at 8 m, DOD	8.4
3	34 M	Gluteus	27	Y, R0	—	Nil (declined Rx)	—	—	Alive, NED	37.3^a^
4	39 M	Upper back	100	Y, R0	50 Gy, 25#	NA+ Adj: VDC/IE	Nil (rapid progression)	Lung, pleura, nodal	Distant R at 13 m, DOD	16.0
5	43F	Chest wall	51	Y, R0	58 Gy/ 29# (with ifosfamide)	Adj: Epirubicin Ifosfamide	—	—	Alive, NED	27.2^a^
6	30F	Neck	60	Y, 0.4 mm	66 Gy, 33#	Adj: VDC/IE	—	—	Alive, NED	54.3^a^
7	24F	Thigh	U	Y, R0	—	NA: VDC/IE	—	—	Alive, NED	65.1^a^
8	31F	Retroperitoneum	150	N	45 Gy, 25#	VIDE x6, VAI x2	IT, etoposide	Lung	Distant R at 11 m, DOD	19.4
9	27 M	Brain	65	Y, U	Brain 36Gy/20#, CSI 23.4Gy/ 13#	Adj: Cisplatin, vincristine, lomustine cyclophophamide	Nil (rapid progression)		Local & distant R at 6 m, DOD	7.7
10	13 M	Thigh	60	Y, R0	55.8 Gy/ 31#	NA + Adj: AI	Nil	Lung, brain, bone	Distant R at 36 m, DOD	41.2
11	27 M	Groin	85	Y, R0	—	—	—	—	Alive, NED	23.4^a^
Advanced disease at diagnosis										
12	22 M	Thigh	115	Y	50 Gy in 20# to pelvis	—	VID, VAC/IE	Lung	DOD	16.6
13	56F	Iliac wing	122	Y	36 Gy in 12# to pelvis	—	Doxorubicin, VDC/IE, pembrolizumab	Lung, brain	DOD	7.6
14	39 M	Para‐testicular	50	Y	—	—	VDC/IE	Lung, bone	ANED	28.1^a^ ^y^
15	26 M	Lung/ pleura	Diffuse	N	20 Gy in 5# to hemithorax	—	VAC/IE	Lung, pleura, nodal	DOD	9.8
16	27 M	Thigh	59	N	55 Gy in 25#	—	VDC/IE, TC, IT	Lung	AWD	14.0
17	14F	Lung	120	Y	15 Gy in 10# VMAT to whole lung, 36 Gy in 18# to tumour bed	—	Ifosfamide, pazopanib	Lung, soft tissue, brain	DOD	12.7
18	21F	Thigh	60	Y	20 Gy in 5# to lung	—	Nil (declined treatment)	Lung	DOD	4.7

Abbreviations: Adj, adjuvant; AWD, alive with disease; DOD, died of disease; F, female; Gy, grey; M, male; M, month; N, no; NACT, neo adjuvant; NED, no evidence of disease; P, palliative; R0, microscopic complete resection; R, recurrence; Rx, treatment; U, unknown; Y, yes.

Treatment regimens as detailed above.

^a^ Censored.

All tumours represented high grade round cell undifferentiated sarcoma with sheets of cells with variable lobulation in a fibrous stroma with some nuclear pleomorphism and vescicular nuclei with prominent nucleoli. Mitotic activity was brisk, necrosis was common and in areas a myxoid stroma was noted. CD99 expression by immunohistochemisty (IHC) was noted in all cases with a patchy, variably diffuse and focally membranous appearance. WT‐1 nuclear expression was noted in 79% of available cases; CD99 and WT‐1 results were not available for five and four cases respectively. ETV4 IHC was positive in three out of three cases tested. *EWSR1*‐rearrangement was tested for by FISH in 16 patients and was negative in all.

*CIC*‐rearrangement was confirmed by FISH in all cases. *DUX4* was identified as the *CIC*‐rearrangement partner in three patients. *CIC‐FOX04* (case 16) and *CIC‐CREBBP* (case 8) were identified in two patients through genomic sequencing. The rearrangement partner was not identified or available for 13 patients. Molecular profiling by next generation sequencing had been completed in six patients. No actionable variants were identified. A number of variants of uncertain significance were identified including *ETV4* splice variants in two patients and an *FGF4* variant in one patient. Tumour mutational burden (TMB) was reported as low in five patients, noting these were generated across different platforms without defined intervals of significance.

### 3.2 Outcomes

**FIGURE 1:**
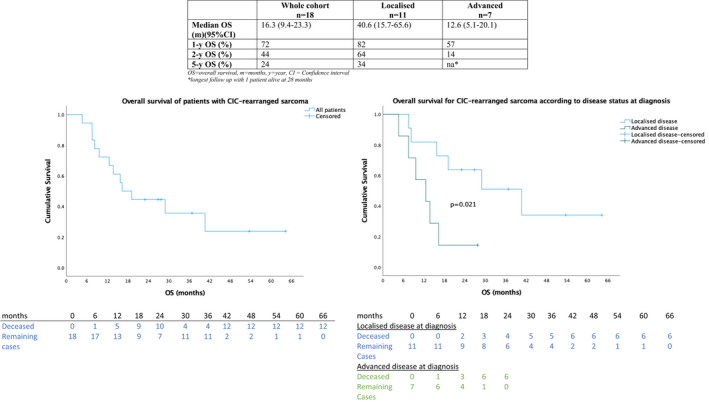
Kaplan–Meier curves of overall survival of CIC‐rearranged sarcoma

**FIGURE 2:**
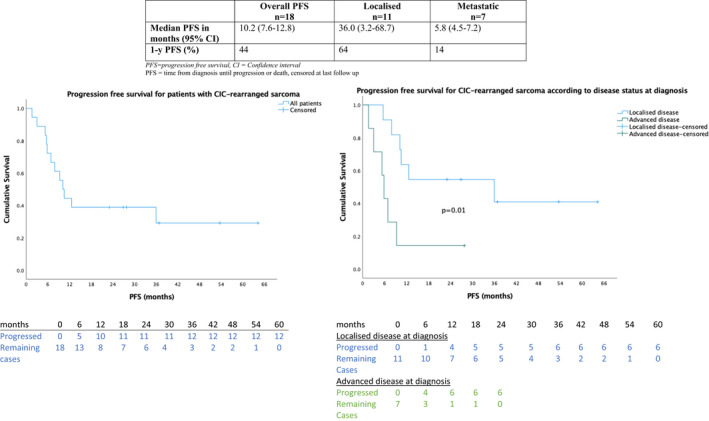
Kaplan–Meier curves of progression free survival of CIC‐rearranged sarcoma

Median follow‐up was 36.8 months (range 4.6–64.2). As illustrated in Figure 1, median OS from diagnosis was 16.3 months (95% CI 9.4–23.3) with 1‐, 2‐ and 5‐year OS of 72%, 44% and 24% respectively. As illustrated in Figure 2, median PFS from diagnosis for all patients was 10.2 months (95% CI 7.6–12.8) with a 1‐year PFS of 44%.

At the last follow up, of the 11 patients with localised disease, 5 (45%) patients are alive without evidence of disease and 6 (55%) developed metastatic disease with a median time to metastases of 10.5 months. Three patients (27%) developed local recurrence with a median time to local recurrence of 5.7 months. The median time to local or distant progression overall was 10.5 months (range 3.0–36.5) and all of these six patients are now deceased. The median PFS from diagnosis was 36.0 months (95% CI 0.2–71.8), median OS was 40.6 months (15.7–65.6) and the 1‐, 2‐ and 5‐year OS were 82%, 64% and 34% respectively.

Of seven patients with advanced disease at diagnosis, six are deceased and one patient remains in a complete response 27.3 months following the start of chemotherapy and 20.8 months without active therapy. In patients with advanced disease, with most receiving multi‐modality therapy upfront, median PFS from diagnosis was 5.8 months (95% CI 4.5–7.2). Median OS was 12.6 months (95% CI 5.1–20.1) and 1‐year OS was 57%.

### 3.3 Localised disease management

Of 11 patients with localised disease at diagnosis, nine (82%) underwent surgery (all had R0 resections), eight (73%) patients received radiotherapy to the primary site and eight (73%) patients received chemotherapy.

Surgery was the initial modality of treatment in six of nine cases. Surgery was not undertaken in two cases where primary tumours were deemed unresectable after inadequate response to induction chemotherapy. Of two patients who had surgery alone, neither have developed local or distant recurrence, 37.3 and 26.2 months from surgical resection respectively.

Radiotherapy was undertaken post operatively in six patients (mean 58Gy in 30 fractions) of whom two developed local (and distant) recurrence. Definitive dose radiotherapy was utilised after chemotherapy in two cases (45Gy in 25 fractions and 50Gy in 25 fractions respectively) which were deemed unresectable. Both patients developed metastatic disease with one patient developing local recurrence also.

Chemotherapy was the initial modality of therapy in five patients and adjuvant chemotherapy was delivered after surgical resection in three patients. Where evaluable, best radiological response to neoadjuvant chemotherapy was partial in all five patients. Treatment response in the resection specimen after neoadjuvant chemotherapy was available for three patients. Responses of more than 90% therapy‐related changes (case 10), 50% necrosis (case 7), and less than 50% necrosis (case 4) were noted. Cases 10 and 4 both received pre‐operative chemotherapy and radiotherapy, and both developed disease recurrence at 36.5 months and 12.8 months respectively. Case 7 received only pre‐operative chemotherapy and remains disease free 65.1 months from diagnosis. Among the six patients who underwent resection and received chemotherapy, 3 patients (50%) developed distant recurrence between 6 and 36 months.

### 3.4 Advanced disease management

Of seven patients with advanced disease at diagnosis; five underwent resection of the primary lesion (four upfront, one after pre‐operative chemotherapy), six received systemic therapies (three upfront, three commenced post resection), and six received palliative dose radiotherapy (one after surgery, five after chemotherapy) to the primary site (median 43Gy in 16 fractions, treatment displayed in Table 1). Radiotherapy was used for palliative treatment of metastases including a case of spinal cord compression.

### 3.5 Systemic treatments

Systemic treatments and outcomes are summarised in Table 2. All patients with localised disease, who received chemotherapy, were treated with multi‐agent regimens which were predominantly Ewing sarcoma based protocols. In advanced disease, durable responses to multiple systemic therapies were limited. The median duration of systemic treatment response, in advanced disease when 14 regimens were analysed, was 2.1 months (range 0.7–27.3). The best response to treatment in advanced disease was partial in all except one patient with oligometastatic disease who had a sustained complete response to seven cycles of vincristine, doxorubicin, cyclophosphamide alternating with ifosfamide and etoposide (VDC/IE) chemotherapy. Complete response is ongoing 27.3 months from commencing treatment, and 20.8 months from last systemic treatment. One patient received pazopanib with disease progression occurring within 30 days. One patient, with a TMB of 4.5 mutation/mb, received a single cycle of pembrolizumab with disease progression and death occurring within 30 days.

**TABLE 2:** Systemic treatment use in 19 cases of CIC‐rearranged sarcoma, organised by use single or multi‐modality approach and by systemic treatment type

Case ID	Disease status	Line of treatment	Medication/protocol	Number of cycles	Best response	Treatment status	Reason treatment discontinued	Time to progression (months)
Chemotherapy as single modality of therapy								
12	A	1	VID	6	PR	Ceased	PD	4.1
12	A	2	VDC/IE	3	PD	Ceased	PD	3.1
13	A	2	VDC/IE	6	PR/SD	Ceased	PD	3.9
14	A	1	VDC/IE	7	CR	Ceased	Completed	27.3* months
15	A	1	VDC/IE	8	PR	Ceased	PD	5.6
13	A	1	Doxorubicin	1	PD	Ceased	PD	0.8
1	A	1	AC	3	PD	Ceased	PD	2.9
1	A	2	GD	3	PD	Ceased	PD	2.2
16	A	2	TC	3	PD	Ceased	PD	2.1
16	A	3	IT	1	PD	Ceased	PD	0.8
1	A	3	IT	2	PD	Ceased	PD	1.5
8	A	2	IT	1	NE	Ceased	Toxicity	NA
8	A	3	Etoposide	3	PD	Ceased	PD	2.1
13	A	3	Pembrolizumab	1	PD	Ceased	PD	0.7
17	A	2	Pazopanib	1	PD	Ceased	PD	0.8
Chemotherapy as part of multi modality therapy (with surgery +/− radiotherapy)								
10	L	1	AI	5	Metastatic relapse	Ceased	Completed	35.8
17	A	1	AI	6	PR	Ceased	Completed	8.6
2	L	1	VDC/IE then VCDE from cycle 4	6	Metastatic relapse	Ceased	Completed	6.5
4	L	1	VDC/IE	3	Metastatic relapse	Ceased	Completed	10.3
8	L	1	VIDEx6, VAIx2	8	Metastatic relapse	Ceased	Completed	10.2
16	A	1	VDC/IE	7	PR	Ceased	Completed, PD	6.0
9	L	1	Cisplatin, vincristine, lomustine then cyclophosphamide	U	Metastatic relapse	Ceased	Completed	5.0
6	L	1	VDC/IE	7	Disease free	Ceased	Completed	—
7	L	1	VDC/IE	7	Disease free	Ceased	Completed	—
5	L	1	Epirubicin ifofamide, chemo‐radiation with ifosfamide	7	Disease free	Ceased	Completed	—

Abbreviations: A, advanced; CR complete response; L, localised; PD progressive disease; PR, partial response; SD stable disease.

Treatment regimens as detailed above.

## 4 DISCUSSION

*CIC*‐rearranged sarcoma is a rare and only recently recognised distinct entity, with a paucity of published literature. Our study confirms the unique characteristics and poor prognosis of *CIC*‐rearranged sarcoma. To the best of our knowledge, we present the first clinical series to comprehensively detail individualised systemic treatment use and to summarise available literature.

We report that although these tumours have a predilection to arise in soft tissue, they can present as primary lesions in other sites such as bone or brain. The latter two have been detailed only infrequently.^5^, ^6^, ^7^ This cohort confirms these tumours arise in young adults, with 78% of cases in this series arising in those aged 20–40 years. The majority of cases appear to arise in patients aged 40 years and below in available case series (59%–71% of patients).^6^, ^7^, ^8^ However, a wide age range from 6 to 83 years has been described.^2^, ^8^

The prognosis of patients with *CIC*‐rearranged sarcomas was poor and consistent with previous reports. In this study, median OS for the overall cohort was 16.3 months with the 2‐ and 5‐year OS of 44% and 24% respectively. Median OS was 40.6 months in those presenting with localised disease at diagnosis and only 12.6 months in those presenting with advanced disease. Disease recurrence occurred in 55% of those presenting with localised disease at diagnosis. In the largest available series of 115 cases, of which clinical follow up was available for 57 patients, Antonescu et al. report 2 and 5‐year OS rates of 59% and 49% respectively.^2^ Yoshida et al. reviewed 20 cases and reported a median OS of 12 months, an estimated 5‐year OS of 17%, and detailed 13 of 20 (65%) patients to be deceased secondary to disease 3–19 months after diagnosis.^6^ Brady et al. detailed 12 cases of *CIC*‐rearranged sarcoma of which three patients (25%) died between 4 and 19 months of initial presentation.^5^

The usual management for localised disease in this series was surgical resection, with peri‐operative chemotherapy using Ewing sarcoma‐based regimens and adjuvant radiation, after which 55% developed metastases at a median of 10.5 months, and 27% failed locally at a median of 5.7 months. Long‐term disease‐free survival has been achieved with both multi‐modality therapy and with resection alone.

*CIC*‐rearranged sarcomas consistently appear to be less chemo‐sensitive than Ewing sarcomas. This is illustrated by the high frequency of relapse in localised disease and short durations of treatment response in the advanced setting. In this series, and available literature, partial responses to systemic therapy occur (Table 4). However, our findings demonstrated that responses are short‐lived, suggesting rapid development of treatment resistance. Systemic treatment survival outcomes, and treatment response, in available literature are summarised in Tables 3 and 4.

**TABLE 3:** Literature summary of overall survival of 42 patients with systemic treatment use. Organised by first line treatment regimen and localised or advanced disease at diagnosis

Localised/advanced disease at diagnosis	Regimen	Author	ID	Disease Status	Follow up time or survival time from date of diagnosis (months)	Survival rate at 6 months	Survival rate at 12 months
**Localised**	**Ewings‐based 1st line regimen**					**100%**	**45%**
	VIDE, VAI	Brcic 2020	2	NED	30†		
	VIDE	Brcic 2020	5	DOD	8		
	VIDE	Brcic 2020	6	DOD	6		
	“Ewing protocol” (neoadjuvant)	Mangray 2018	2	DOD	6		
	VDC (adjuvant)	Mangray 2018	3	AWD	8†		
	CODE, VDC‐I	Yoshida 2016	10	AWD	6†		
	*as per* Table 1	*Connolly 2022* ^*^	*2,4,6–8*	*—*	*—*		
**Localised**	**Anthracycline‐containing soft tissue sarcoma‐based 1st line regimen**					**100%**	**67%**
	AI	Brcic 2020	1	DOD	10		
	AI (adjuvant)	Mangray 2018	1	NED	36†		
	AI + dacarbazine	Yoshida 2016	8	NED	14†		
	AI	Yoshida 2016	15	NED	7†		
	*as per* Table 1	*Connolly 2022* ^*^	*5,10*	*—*	*—*		
**Advanced**	**Ewings‐based 1st line regimen**					**100%**	**59%**
	VDC × 3, VAC × 3, IE, trabectedin	Kimbara 2021	1	DOD	13		
	VIDE, IT + Bevacizumab	Brcic 2020	3	DOD	15		
	VAIA, pazopanib	Brcic 2020	4	DOD	14		
	AI/VAC, gemcitabine + paclitaxel, ifosfamide (high dose)	Sedighim 2020	1	DOD	15		
	VDC/IE × 7, VAI × 2, SCT, pazopanib, IT	Nakai 2019	1	DOD	16		
	VDC/IE	Yoshida 2016	6	DOD	8		
	VAC, VDC/IE, TC, paclitaxel	Yoshida 2016	9	DOD	6		
	VDC/IE, cisplatin/irinotecan, AI	Yoshida 2016	12	DOD	10		
	VAC/ VDC/IE, irinotecan, TC	Yoshida 2016	14	DOD	11		
	VDC/IE, TC	Yoshida 2016	16	ANED	74†		
	VDC, I	Yoshida 2016	17	DOD	12		
	VDC/IE, VACA, ICE	Yoshida 2016	18	DOD	9		
	VDC/IE, VIDE	Yoshida 2016	19	AWD	7†		
	*as per* Table 1	*Connolly 2022* ^*^	*13,15–17*	*—*	*—*		
**Advanced**	**Anthracycline‐containing soft tissue sarcoma‐based 1st line regimen**					**75%**	**50%**
	AI	Yoshida 2016	1	DOD	8		
	AI, VAIA, gemcitabine	Yoshida 2016	3	DOD	19		
	AI, Pazopanib	Yoshida 2016	4	DOD	13		
	AI, Gemcitabine/ Docetaxel	Yoshida 2016	5	DOD	14		
	Doxorubicin	Yoshida 2016	7	DOD	4		
	Doxorubicin, dacarbazine	Yoshida 2016	11	DOD	3		
	AI, pazopanib	Yoshida 2016	20	DOD	18		
	*as per* Table 1	*Connolly 2022* ^*^	*14*	*—*	*—*		
	†remains alive						

Abbreviations: ANED, alive no evidence of disease; AWD, alive with disease; DOD, died of disease.

Treatment regimens as detailed above.

^*^ Reflects patients from this series.

**TABLE 4:** Literature summary of 38 disease response in 38 patients to systemic treatment use

Regimen	Author	Total	CR/PR	SD	PD
		*n*	*n* (%)	*n* (%)	*n* (%)
VDC/IE	*Connolly 2022* ^*^	*5*	4 (80)	0	1 (20)
Other vincristine based regimen	*Connolly 2022* ^*^ *, Kimbara 2021* ^12^ *, Italiano 2011* ^9^	*7*	3 (43)	3 (43)	1 (14)
Anthracycline single/doublet regimen	*Connolly 2022* ^*^, Ricker 2020^15^, Choi 2013^18^, Italiano 2011^9^	*8*	3 (38)	1 (13)	4 (50)
IE, etoposide	*Connolly 2022* ^*^, Kimbara 2021^12^, Choi 2013^18^	*4*	1 (25)	0	3 (75)
Ifosfamide (high dose)	*Sedighim 2020* ^19^ *, Choi 2013* ^18^	*2*	1 (50)	0	1 (50)
Taxane based doublet (gemcitabine docetaxel, gemcitabine paclitaxel, TC)	*Connolly 2022* ^*^ *, Sedighim 2020* ^19^	*3*	0	0	3 (100)
Irinotecan temozolamide	*Connolly 2022* ^*^ *, Nakai 2019* ^20^	*3*	0	0	3 (100)
Trabectedin	*Kimbara 2021* ^12^	*1*	0	0	1 (100)
Pazopanib	*Connolly 2022* ^*^ *, Nakai 2019* ^20^	*3*	0	0	3 (100)
Pembrolizumab	*Connolly 2022* ^*^ *, Ricker 2020* ^15^	*1*	0	0	1 (100)^a^
Phase I B7H3‐targetted antibody MGA‐271	*Ricker 2020* ^15^	*1*	0	0	1 (100)

Abbreviations: CR, complete response; PD, progressive disease; PR, partial response; SD, stable disease.

Treatment regimens as detailed above.

^a^ Mixed response cited by Ricker et al. though details development of new metastases followed by PD.

^*^Reflects patients from this series.

When considering the treatment approach for localised disease, the authors would advocate consideration for initial resection, rather than neo‐adjuvant therapy, given the efficacy of chemotherapy in the localised setting is unclear and delayed resection may increase metastatic risk. One patient (case 2) in this series developed local progression while receiving initial systemic therapy and progression on pre‐operative chemotherapy has also been cited by Italiano et al..^9^ This viewpoint is further supported by Antonescu et al. who found patients treated with neoadjuvant chemotherapy (*n* = 22) showed an inferior survival compared with patients managed by surgery first (*n* = 29) (*p* = 0.025). It was noted, however, that patients selected for neoadjuvant therapy had a larger tumour size (*p* < 0.0001) compared with patients who were managed by surgery first which may have confounded the findings.^2^ When considering the utility of neo‐adjuvant chemotherapy to down‐stage disease and improve surgical morbidity, partial responses to therapy were observed. However, no complete responses of localised disease occurred in this series nor are detailed in the literature.

When reviewing neo‐adjuvant treatment response, three patients were available for evaluation; pathological response of more than 90% necrosis (case 10) and less than 50% necrosis (case 4) were observed after neo‐adjuvant chemotherapy and radiotherapy, and, 50% necrosis (case 7) after pre‐operative chemotherapy alone. The utility of pathological response in *CIC*‐rearranged sarcoma as a surrogate marker of prognosis, as has been established in Ewing sarcoma and osteosarcoma,^10^, ^11^ has not been shown; case 11 who had a superior pathological response had a long disease‐free interval (36 months) although ultimately developed fatal disease recurrence and case 8, who had an inferior response, achieved long term disease control without recurrence (disease free at 59 months). This is in keeping with Antonescu et al. who found no correlation between survival and the degree of response when 10 patients were analysed including 3 patients who had achieved greater than 90% therapy‐related change.^2^

In advanced disease, durable responses to systemic therapy appear to be limited (Tables 2 and 4) with no agent or regimen demonstrating clear efficacy. One exceptional response to VDC/IE (case 14) was seen. Yoshida et al. also detail a similar exceptional response, of a duration of at least 79 months, with treatment including VDC/IE, topotecan and cyclophosphamide.^6^ Sequential VDC followed by second‐line IE chemotherapy has been used, providing disease control for approximately 12 months.^12^ Italiano et al. reported a complete response of metastatic disease to doxorubin and ifosfamide.^9^ The duration of response, however, was not reported. In available literature, OS in advanced disease appears to be similar whether multi‐agent Ewing based regimens (1‐year OS 59%), or soft tissue anthracycline doxorubicin based regimens (1‐year OS 50%), are used in the first line (Table 3). The small numbers included when considering these survival statistics, in particular those who have received anthracycline containing soft tissue sarcoma‐based treatment, must however be considered.

Our series adds to limited reports of molecularly targeted therapies and immunotherapeutics in *CIC*‐rearranged sarcoma.^7^, ^13^, ^14^, ^15^ Unfortunately, treatment efficacy was not demonstrated in our patients treated with pazopanib and pembrolizumab, with rapid disease progression occurring in both. Of patients who underwent genomic sequencing, no actionable mutations nor therapeutic options were identified. There is a paucity of individualised systemic therapy outcome (progression free survival) evidence in current literature for all treatments and the efficacy of non‐anthracycline‐containing soft tissue sarcoma regimens, molecularly targeted therapies and immunotherapy remains to be determined.

Our study has several limitations including its retrospective nature, small sample size, and potential for selection bias. A centralised pathological review has not been undertaken, however, there is a high level of certainty of diagnosis as all diagnoses were made at specialised sarcoma centres and *CIC*‐rearrangement has been confirmed with FISH in all cases.

In future, international collaboration will be required to determine therapeutic approaches and to develop consensus guidelines. Further research is needed to better understand the unique disease biology of this entity, to develop novel therapeutics, and to identify biomarkers of disease response, especially in exceptional responders. Access to early phase clinical trials, and translational research, will be key in identifying efficacious novel agents from which *CIC*‐specific clinical trials could follow. Although challenging, subtype‐specific trials of ultra‐rare sarcomas are possible through international collaboration as evidenced by the ‘CASPS’ trial of cediranib for alveolar soft part sarcoma^16^ and the phase 2 basket trial of tazemetostat for epithelioid sarcoma.^17^

**In conclusion,** in this series *CIC‐*rearranged sarcoma affected young adults with a high incidence of presenting with, or developing, metastatic disease. Prognosis overall was poor with a median OS of 16.3 months. Usual management for localised disease was surgical resection, chemotherapy with Ewing‐based regimens, and adjuvant radiation, after which 55% developed metastases at a median time to progression of 10.5 months. In advanced disease, durable systemic therapy responses occurred infrequently with a median duration of systemic treatment response of 2.1 months. Radiotherapy to the primary site was used frequently in localised and advanced disease. Further research through international collaboration is needed to establish optimum treatment approaches for localised and advanced disease.

## CONFLICTS OF INTEREST

The authors declare that there is no conflict of interest regarding the publication of this article.

## AUTHOR CONTRIBUTIONS

Conception and design: EC, AH; Financial support: none; Administrative support: EC, AH;

Data analysis and interpretation: EC, VB, JW, PG, MS, FB, AH;

Provision of study materials or patients, collection and assembly of data, manuscript writing, and

final approval of manuscript: all authors.

## ETHICS STATEMENT

Data collection and usage for this study was approved by the Sydney Local Health Human Research Ethics Committee (X17‐0340).

## Data Availability

The data that support the findings of this study are available from the corresponding author upon reasonable request.
